# Gaussian modelling for operator-independent and threshold-free volumetric segmentation of phase sensitive inversion recovery late gadolinium enhanced images

**DOI:** 10.1186/1532-429X-13-S1-P43

**Published:** 2011-02-02

**Authors:** Stefan K Piechnik, Erica Dall'Armellina, Vanessa M Ferreira, Matthew D Robson

**Affiliations:** 1 University of Oxford, Oxford, UK

## Aim

To objectively determine the volume of damage measured by late Gadolinium enhancement (LGE) using threshold-free statistical fit to the distribution of image intensities to reflect the tissue heterogeneity, presence of noise and partial volume effects.

## Background

Infarct size following STEMI carries prognostic significance. Current CMR methods for measuring abnormal myocardium are based on detecting pixels above a certain intensity threshold. This threshold is often defined from a ROI as the mean plus a number (nSD) of standard deviations. This method assumes that the ROI is “normal” and not affected by disease, and is sensitive both to the choice of the ROI and the nSD multiplier. Objective determination of the volume of damage on LGE images may be desirable.

## Material and methods

17 STEMI patients aged 56±9 years underwent 3T CMR imaging within 48h post PCI using a phase-sensitive late-gadolinium enhanced inversion recovery (PSIR) method. Histograms of myocardial signal intensities were calculated in each patient and fitted with a sum of two Gaussians. We calculated the relative volume fraction (Vf_Gauss_) as the area under the Gaussian distribution with the higher peak divided by the total area under both peaks. For comparison, traditional threshold segmentation was performed based on ROI placed in remote myocardium and varying the threshold nSD from 0 to 6, to determine the traditional volume fraction (Vf_nSD_).

## Results

**Figure 1:**
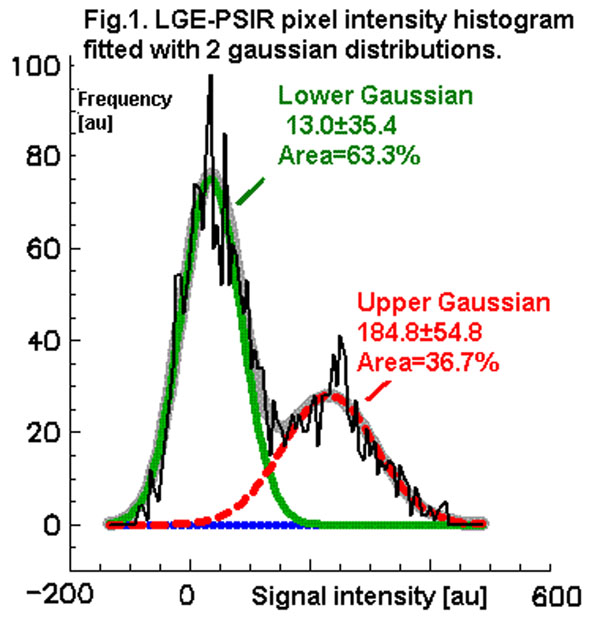
LGE-PSIR pixel intensity histogram fitted with 2 gaussian distributions.

**Figure 2:**
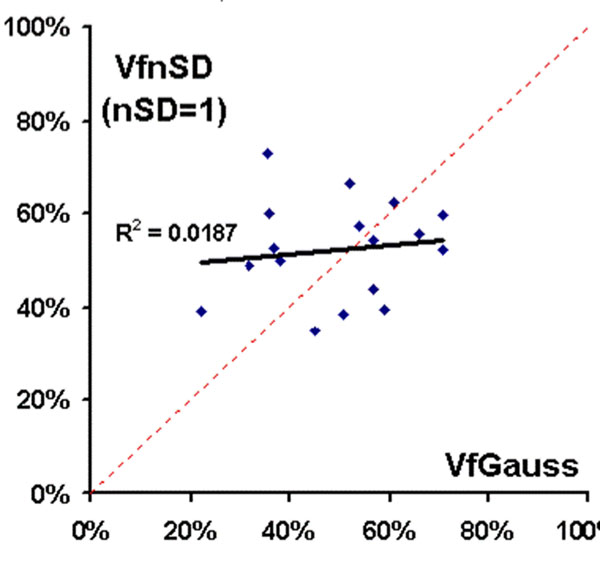
Volume fractions agree for nSD=1, but correlation is weak.

**Figure 3:**
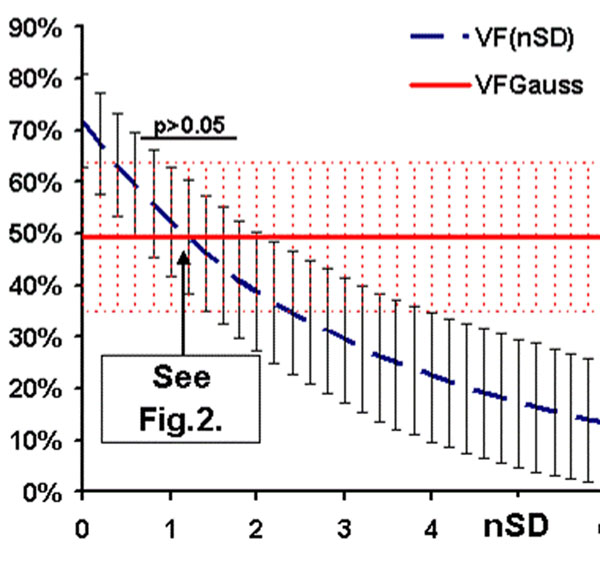
Impact of nSD on traditional volume fraction. VfGauss is a constant.

All LGE histograms were accurately described by just two Gaussian components with a high coefficient of explained variation R^2^=0.89-0.98 (Fig. 1). The lower Gaussian curves were consistently centered near zero (SI = -16±38 scanner units[su]) and relatively narrow in distribution (SD=39±12[su]). The Upper Gaussian had a greater signal intensity (182±89[su]), and a significantly wider distribution (SD=81±34[su], p<0.001) than the Lower Gaussian. Average Vf_Gauss_ was 49±14%, which corresponds best to the traditional Vf calculated by using a threshold of about 1.0SD (Fig.2 Arrow in Fig.3). Traditional Vf_nSD_ strongly depended on nSD as expected, and there was a significant difference between Vf_nSD_ and Vf_Gauss_ (p>0.05) except for nSD=0.8-1.8 (Fig. 3).

## Conclusions

Gaussian modelling splits the signal intensity distribution consistently into two distinct components: the narrow “normal” peak centred about the null point and a more heterogenous “abnormal” hyperintense pixel distribution. This directly leads to lesion volume fractions that are free from arbitrary choice of ROI or any thresholds, which may improve the consistency and objectivity of LGE image analysis.

